# Resistance and Resilience of Sulfidogenic Communities in the Face of the Specific Inhibitor Perchlorate

**DOI:** 10.3389/fmicb.2019.00654

**Published:** 2019-04-02

**Authors:** Magdalena K. Stoeva, Gilbert Nalula, Nicholas Garcia, Yiwei Cheng, Anna L. Engelbrektson, Hans K. Carlson, John D. Coates

**Affiliations:** ^1^Department of Plant and Microbial Biology, University of California, Berkeley, Berkeley, CA, United States; ^2^Energy Biosciences Institute, Berkeley, CA, United States; ^3^Department of Integrative Biology, University of California, Berkeley, Berkeley, CA, United States; ^4^Climate and Ecosystem Sciences Division, Lawrence Berkeley National Laboratory, Berkeley, CA, United States; ^5^Environmental Genomics and Systems Biology Division, Lawrence Berkeley National Laboratory, Berkeley, CA, United States

**Keywords:** perchlorate, specific inhibition, sulfate reduction, chemostat, microbial communities, surface attachment, growth rate

## Abstract

Hydrogen sulfide is a toxic and corrosive gas, produced by the activity of sulfate-reducing microorganisms (SRM). Owing to the environmental, economic and human-health consequences of sulfide, there is interest in developing specific inhibitors of SRM. Recent studies have identified perchlorate as a promising emerging inhibitor. The aim of this work is to quantitatively dissect the inhibitory dynamics of perchlorate. Sulfidogenic mixed continuous-flow systems were treated with perchlorate. SRM number, sulfide production and community structure were monitored pre-, during and post-treatment. The data generated was compared to a simple mathematical model, where SRM growth slows as a result of inhibition. The experimental data supports the interpretation that perchlorate largely acts to suppress SRM growth rates, rendering planktonic SRM increasingly susceptible to wash-out. Surface-attachment was identified as an important parameter preventing SRM wash-out and thus governing inhibitory dynamics. Our study confirmed the lesser depletion of surface-attached SRM as compared to planktonic SRM during perchlorate treatment. Indirect effects of perchlorate (bio-competitive exclusion of SRM by dissimilatory perchlorate-reducing bacteria, DPRB) were also assayed by amending reactors with DPRB. Indeed, low concentrations of perchlorate coupled with DRPB amendment can drive sulfide concentrations to zero. Further, inhibition in a complex community was compared to that in a pure culture, highlighting similarities and differences between the two scenarios. Finally, we quantified susceptibility to perchlorate across SRM in various culture conditions, showing that prediction of complex behavior in continuous systems from batch results is possible. This study thus provides an overview of the sensitivity of sulfidogenic communities to perchlorate, as well as mechanisms underlying these patterns.

## Introduction

Microbial sulfate reduction (SR) by sulfate-reducing microorganisms (SRM) is one of the primary pathways of anaerobic terminal organic matter mineralization, and as such is a determinant of carbon and sulfur cycling in many environments (Hao et al., 1996; Barton and Fauque, 2009; Plugge et al., 2011; Whitman et al., 2014). SR results in the generation of hydrogen sulfide, a toxic gas with adverse human health effects (WHO, 2000). Sulfide production also has significant economic and environmental consequences owing to its corrosive nature (Hao et al., 1996). Roberts et al. (2002) quantified sulfide-induced concrete corrosion rates of up to 5 mm/year in sewer systems. Further, reservoir souring (microbial sulfide production in oil reservoirs) causes a decrease in oil quality, infrastructure corrosion, and reservoir plugging (Gieg et al., 2011). As a result of this economic and industrial importance, SR has been studied with a multiplicity of different tools, and in various systems (Widdel and Bak, 1992; Barton and Fauque, 2009; Plugge et al., 2011; Enning and Garrelfs, 2014).

Concurrent with and central to studies on the ecology of SR, has been the identification and characterization of specific inhibitors of SRM. Specific inhibitors are compounds that disrupt the metabolism of one group of organisms, with little or no effect on the rest of the community (Oremland and Capone, 1988). Such compounds have been used in microbial ecology to uncover electron flow through certain metabolic processes in natural systems (Oremland and Capone, 1988). Additionally, specific inhibitors are of potential value to industrial systems. Postgate (1952) was the first to screen a panel of sulfate analogs in search of specific inhibitors of the sulfate reducer *Desulfovibrio desulfuricans* strain Hildenborough, and identified perchlorate, among other inhibitors (Postgate, 1952). Perchlorate has since been characterized as a competitive inhibitor of the SR pathway (Carlson et al., 2015a). Furthermore, since perchlorate can also serve as an electron acceptor for microbial respiration, it has several indirect mechanisms of inhibition: (1) bio-competitive exclusion, whereby dissimilatory perchlorate-reducing bacteria (DPRB) perform a more energetically favorable metabolism than SR and thus outcompete SRM (Engelbrektson A. et al., 2014; Cheng et al., 2016); (2) potential production of toxic intermediates by DPRB: chlorite, chlorate, or oxygen; and (3) sulfide re-oxidation to insoluble sulfur by DPRB (Gregoire et al., 2014; Mehta-Kolte et al., 2017). Due to its potency and selectivity against all tested SRM, perchlorate has been proposed as a promising compound for the inhibition of sulfide production in oil reservoirs (Engelbrektson A. et al., 2014, 2018; Carlson et al., 2015a; Engelbrektson A.L. et al., 2018).

Much of the current insight on specific inhibition of SR has been gained via pure culture studies (Postgate, 1949; He et al., 2010; Korte et al., 2014, 2015; Carlson et al., 2015a). Although useful, this model organism view is challenging to extrapolate to complex natural communities. On the other hand, several studies have examined the effects of perchlorate in complex systems (Engelbrektson A. et al., 2014, 2018; Carlson et al., 2015a,b; Engelbrektson A.L. et al., 2018). These systems, though highly relevant, are confounded by complexity and variability. Here, we use a simple planktonic, continuous-flow, sulfidogenic undefined community to characterize the relevant biology pertaining to inhibition of SR by perchlorate. This set-up allows for the generation of simple mathematical models and the dissection of inhibitory dynamics, yet retains biological complexity and relevance.

We characterize the effects of perchlorate in terms of the rate and level of inhibition of SR, as well as the rate and level of post-treatment rebound of SR. Empirical data is compared to a simple model, elucidating two factors key to inhibitory dynamics, namely: (1) the dose-response/inhibition curve of SRM against perchlorate and (2) surface-attachment of microbial cells. Surface-attachment, as well as the indirect effects of perchlorate (mediated by DPRB) are explored further, via direct manipulation. Finally, inhibition in a complex community is compared to that in a pure culture, highlighting similarities and differences between the two scenarios. Together, the experiments performed provide a comprehensive illustration of the inhibitory dynamics of perchlorate, and factors that affect it, both in pure culture and in a community setting.

## Materials and Methods

### Chemostat Setup and Operation

Chemostats were custom made from glass bottles equipped with influent and effluent lines and a liquid sampling port (Adams & Chittenden, Berkeley, CA, United States) (Supplementary Figure S1A). Inflow artificial porewater medium (APM) consisted of 20 g/L NaCl, 0.67 g/L KCl, 6.8 g/L N-[Tris(hydroxymethyl)methyl]-2-aminoethanesulfonic acid (TES), vitamins, and minerals (Bruce et al., 1999), as well as salts prepared separately in a concentrated stock and added post-autoclaving (20x: 21.2 g/100 mL MgCl_2_x6H_2_O, 3.04 g/100 mL CaCl_2_x2H_2_O; 50x: 4 g/100 mL NaCl, 5 g/100 mL NH_4_Cl, 0.5 g/100 mL KCl, 0.5 g/100 mL KH_2_PO_4_, 1 g/100 mL MgSO_4_x7H_2_O, 0.1 g/100 mL CaCl_2_x2H_2_O). Yeast extract (2 g/L) or sodium lactate (60 mM) served as the electron donor and carbon source and 20 mM sodium sulfate served as the electron acceptor. Media was flushed with N_2_ to render it anaerobic, and was pumped in from a reservoir bottle (also kept under N_2_) at a set flow rate of approximately 2.6 ml hr^−1^, resulting in a residence time of 48 h. This flow rate was chosen to accommodate slow-growing microorganisms and maintain diversity within the community. Chemostats were kept on a stir plate at 30°C and effluent drained passively such that a constant volume of V = 125 ml was maintained. Replicate chemostats were sterilized, rendered anaerobic (by degassing with N_2_) and inoculated at 10% with a marine sulfidogenic community collected from San Francisco Bay sediment and pre-enriched on yeast extract and sulfate. Post inoculation, chemostats were allowed to reach steady-state (defined as stable sulfide production over approximately 7 days) prior to treatment with perchlorate. Control chemostats were left untreated. Once inhibition stabilized, treatment was ceased and rebound of sulfide was monitored (Supplementary Figure S1B). Sodium perchlorate was added at 20, 50, or 80 mM respectively. Perchlorate and sulfate concentrations were quantified via ion chromatography on a Dionex ICS-1500 using Dionex IonPac AS25 4X250 mm anion-exchange column, with an AG25 4X50 mm guard column and a 36 mM NaOH flow rate of 1 ml/min. Sulfide was measured using a modified Cline assay (Cline, 1969) and read at 660 nm on a Spectramax M2 spectrophotometer (Molecular Devices, Sunnyvale, CA, United States). Optical density was measured at 600 nm on the same instrument.

An initial planktonic culture chemostat experiment was performed using 50 mM perchlorate, where community structure was monitored via 16s rRNA amplicon sequencing (Table 1). A second experiment with 20, 50, or 80 mM perchlorate was subsequently completed to explore the effect of different concentrations of perchlorate. A third experiment assessed increased surface-attachment at 50 mM perchlorate by filling chemostats with glass beads (4.5 mm, Zymo Research, Irvine, CA, United States). A fourth experiment using 20 mM perchlorate with and without DPRB amendment teased apart direct and indirect mechanisms of perchlorate inhibition. DPRB were added as a mixture of 4 strains: *Arcobacter sp.* CAB, *Sedimenticola selenatireduces* CUZ, *Marinobacter vinifirmus* UCB, and *Denitromonas halophilus* SFB-3 (Carlström et al., 2013, 2015, 2016).

**Table 1:** Summary of chemostat experiments.

Run	Purpose	Treatment		Culture
		[perchlorate] (mM)	Additional Factor(s)	
*1: Initial run*	Inhibitory dynamics and effects on community structure	50		Sulfidogenic community
*2: Validation run*	Testing inhibition at various perchlorate concentrations	20, 50, and 80		Sulfidogenic community
*3: Surface-attachment run*	Testing the effect of increased surface attachment	50	Glass beads for increased surface-attachment	Sulfidogenic community
*4: DPRB run*	Testing *indirect* effects of perchlorate	20	+/− DPRB to evaluate indirect effects	Sulfidogenic community
*5: Pure-culture run*	Comparing inhibition in pure culture and in a community context	20, 50		*Desulfovibrio* sp. BMSR

To compare perchlorate inhibitory dynamics of a mixed sulfidogenic community to those of pure cultures, the dominant SRM species was isolated from the community, using methods previously described for SRM isolation (Widdel and Bak, 1992). Briefly, serial dilutions were performed in agar plugs with the media described above, supplemented with a trace amount of ferrous iron (FeSO_4_X7H_2_O) as ferrous iron reacts with sulfide and renders SRM colonies black. Single colonies were picked and cultured in APM. The purity of the isolate, *Desulfovibrio* sp. BMSR (IMG ID 2687453694), was confirmed via 16s rRNA sequencing, using primers 27F and 1525R (Lane, 1991). This organism was then used to inoculate a final (fifth) round of chemostats. Sodium lactate (60 mM), supplemented with casamino acids (5 g/L, Bacto, technical grade), replaced yeast extract as the donor and perchlorate was added at 20 or 50 mM. Optical density at 600 nm (OD600), as well as sulfide, sulfate and perchlorate concentrations were once again monitored. Table 1 provides a summary of the five chemostat experiments.

### Community Analyses

Chemostats were sampled for planktonic and surface-attached communities: 1 ml of culture was centrifuged (5 min at 13 000 rpm) and the resulting pellet was immediately stored at −80°C. DNA was extracted using a DNeasy Powersoil HTP 96 kit (Qiagen), following manufacturer’s instructions. For surface-attached communities, 15–20 glass beads were collected and DNA was extracted immediately using a DNeasy PowerBiofilm Kit (Qiagen). DNA concentrations were quantified via a Quant-iT HS Kit (Thermo Fisher Scientific, Waltham, MA, United States), on an Infinite M1000 Pro microplate reader (Tecan), following manufacturer’s instructions.

Amplicon sequencing of the 16S rRNA HV region V4 was conducted using primer pair Miseq 16S F and Miseq 16S R (A519F and Bakt_805R, modified to include Illumina adapters) (Klindworth et al., 2013; Carlström et al., 2016). Libraries were prepared as per the Illumina 16S Metagenomic Sequencing Library Preparation protocol. For analysis where > 96 samples were multiplexed, the Nextera XT Index Kit v2, sets A-D (Illumina) was used. Samples were quantified (Quant-iT), normalized using a Biomek XF^P^ liquid handling robot, and pooled. Sequencing was performed at the UC Davis Genome Center (Davis, CA, United States) on a Miseq (Illumina Miseq Reagent Kit V2). Demultiplexed samples were analyzed according to the Mothur Miseq SOP (Schloss et al., 2009; Carlström et al., 2016). Briefly, chimeras were removed using UCHIME (Edgar et al., 2011) and remaining sequences were screened based on an expected length of approximately 287 bp. Sequences were then clustered at the 97% similarity level and classified using the RDP and SILVA databases. The resulting OTUs were used to generate an OTU table across samples. Abundances were normalized to total amplicon counts per sample. The proportion of SRM was calculated as the combined relative abundance of the *Desulfovibrio*, *Desulforhopalus*, *Desulfocurvus*, and *Desulfomicrobium* genera. No other known SRM were identified. Primer 7 (Clarke and Gorley, 2015) was used to square-root transform normalized abundance data and generate a Bray Curtis similarity matrix, which was in turn used to create non-metric multidimensional scaling (nMDS) plots. SimProf and SIMPER were used to identify significant groupings and to determine OTUs contributing to differences between groupings (Clarke, 1993). Significance of SIMPER-identified OTUs was confirmed using ANOVA and Sidak’s multiple comparison test. All raw sequencing data is available in the sequence read archive (SRA) and all final merged 16s rRNA reads are available in GenBank; both datasets are under the BioProject accession PRJNA521657.

SRM were also quantified using qPCR on the *dsrA* gene (a specific marker of dissimilatory SRM). The *dsrA* gene was PCR amplified with primers DSR1-F+ and DSR-R (Kondo et al., 2004) and cloned into a TOPO cloning vector (TOPO TA Kit, Invitrogen), according to manufacturer’s instructions. The vector was quantified (Quant-iT) and used to generate standard curves (300–3000000 copies). Both SYBR and Taqman workflows were used, as previously described (Spence et al., 2008; Bourne et al., 2011; Carlson et al., 2015b). All samples and controls were run in triplicate on a StepOnePlus RealTime PRC System (Applied Biosystems). Negative controls and the size of the PCR amplicon were confirmed via gel-electrophoresis. *dsrA* copy number was normalized to total ng gDNA.

### Mathematical Model Construction

A simple mathematical model was developed according to basic chemostat theory (Smith and Waltman, 1995), which posits that in a well-mixed system, the resident population increases proportional to a maximum growth rate constant and decreases proportional to the washout (flow rate). The population is limited by an undetermined factor, represented in this case by the carrying capacity, *K*, of the system. Hence, at steady-state, growth of SRM can be modeled as

(1) $$\frac{\text{dN}}{\text{dt}}=\text{μN}\left(1-\frac{\text{N}}{\text{K}}\right)-\frac{\text{N}}{\text{V}}\text{F}$$

Where N is the SRM population, μ is the growth rate, K is the carrying capacity of the ‘environment’ toward SRM, V is the volume of the chemostat and F is the flow rate.

Perchlorate inhibition is assumed to affect the growth rate of SRM, which is consistent with previously published work suggesting that the main mechanism by which SRM overcome perchlorate inhibition is by upregulating the SR pathway (Carlson et al., 2015a). Perchlorate concentration in the chemostat (P) is modeled based on the following differential equation:

(2) $$\frac{\text{dP}}{\text{dt}}={\text{F}}_{\text{Pin}}-\frac{\text{P}}{\text{V}}\text{F}$$

where F_Pin_ is the rate of perchlorate entering the system (equal to the perchlorate concentration in the media reservoir multiplied by the flow rate, F). Perchlorate leaves the chemostat proportional to its concentration in the chemostat ($\frac{\text{P}}{\text{V}}$) at time t multiplied by the flow, F. Solving this equation then allows for the determination of P through time.

Dose-response curves connect the concentration of perchlorate in the chemostat at a given time, to μ, via the parameter y. The equation governing perchlorate inhibition is:

(3) $$\text{y}=\frac{1}{1+10^{\left(\text{LogIC}50-\text{log}[\text{P}])\;(\text{HillsSlope}\right)}}$$

Where IC50 denotes the concentration inhibiting 50% of SRM, and HillSlope describes the steepness of the slope of the dose-response/inhibition curve (refer to the Measuring Inhibition section of the Methods for the empirical determination of these parameters). Thus, y is a fraction representing the effect of a specific concentration of P. As the model assumes that the only effect of perchlorate is a change in the growth rate, y multiplies by μ_max_, to give the actual growth rate as a function of perchlorate concentration and time. See Model Parameterization and Validation below for parameter values for equation (3).

Finally, growth was modeled additively as a combination of planktonic SRM (N_p_ ) and surface-attached SRM (N_A_), with each population being subject to its own carrying capacity, K_p_ and K_A_ respectively:

(4) $$N_{t}=N_{p}+N_{A}\;(4a)$$

(5) $$\frac{{\mathrm{dN}}_{p}}{\mathrm{dt}}=\text{μ}N_{p}\left(1-\frac{N_{p}}{K_{p}}\right)-\frac{N_{p}}{v}F\;(4b)$$

(6) $$\frac{{\mathrm{dN}}_{A}}{\mathrm{dt}}=\text{μ}N_{A}\left(1-\frac{N_{A}}{K_{A}}\right)\;(4c)$$

and from Equation (3), μ = μ_*max*_ × *y*.

The initial model output, *N_t_*, was converted to sulfide concentration, [*sulfide*], for easier comparison to experimental data, using the regression of measured sulfide and measured *N_t_*.

### Measuring Inhibition

Our current understanding of perchlorate inhibition implies a non-biocidal effect of perchlorate on SRM. To confirm this, triplicate *Desulfovibrio* sp. BMSR cultures were incubated in APM with 20 mM sulfate and with one of: (i) no lactate and no perchlorate; (ii) no lactate and 50 mM perchlorate; or (iii) 60 mM lactate and 50 mM perchlorate. Samples were taken at 24 h and mixed with LMT agarose (1%), SYTO 9 (1x, Thermo Fisher) and propidium iodide (8 μg/mL, Thermo Fisher). Cells were imaged using a Zeiss AxioImager M2 at the Berkeley Imaging Facility. ImageJ was used to merge images from different filters and cells were counted manually. Statistical analysis was performed in Graphpad Prism.

Perchlorate inhibition of SR was quantified via dose-response/inhibition curves, generated as previously described (Carlson et al., 2015b). Briefly, 96 or 384-well plates (Costar, Thermo Fisher Scientific) containing anaerobic media and a horizontal gradient of perchlorate concentrations, from 0 to 500 mM (in a serious of twofold dilutions) were made. These gradient plates were inoculated in an anaerobic glove bag (Coy) with culture (pure or mixed community) at an OD of 0.02, sealed, and incubated anaerobically at 30°C for 48–72 h. Growth (OD600) and sulfide (cline assay) were quantified on an Infinite M1000 Pro microplate reader (Tecan). Cline assay was performed in HT using a Biomek XF^P^ liquid handling robot. Data generated was normalized to a control and used to construct dose-response curves in GraphPad Prism 7 (GraphPad Prism Software, Inc., La Jolla, CA, United States) using the “Dose-Response – Inhibition, log(inhibitor) vs. normalized response – variable slope” function. Various donors for growth were used: sodium lactate (40 mM), sodium pyruvate (20 mM), sodium fumarate (20 mM), sodium succinate (20 mM), glucose (30 mM) and yeast extract (2 g/L). Dose-response/inhibition curves were also generated from growth-curve data. Briefly, triplicate anaerobic tubes were inoculated under various perchlorate concentrations and either/and OD600 (measured on a Spectronic 20D+) or sulfide (measured via cline assay) were monitored. Data generated was used to construct growth curves and calculate growth rates. Growth rates were normalized to the control growth rate and used to construct dose-response/inhibition curves, as described above. IC50 and Hillslope values determined were used in the model described above.

## Results

### Inhibitory Dynamics of Perchlorate in a Sulfidogenic Chemostat Community

**FIGURE 1:**
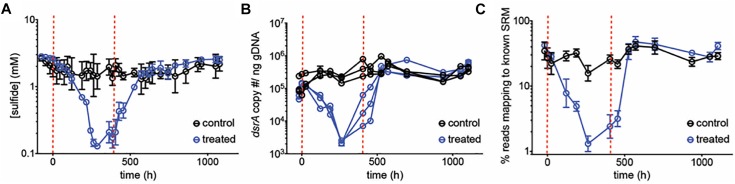
**(A)** Average sulfide production, **(B)** *dsrA* copy number per ng genomic DNA and **(C)** percentage of reads mapping to known SRM, based on 16S amplicon sequencing, over time for control chemostats (black circles) and chemostats treated with 50 mM perchlorate (blue circles). Dashed red lines indicate start and stop of treatment. Error bars represent SD from 3 replicates, except in **(B)** where replicates are graphed independently.

To test the inhibitory dynamics of sulfidogenesis in the presence of perchlorate, a mixed sulfidogenic community was inoculated into triplicate chemostats, treated with 50 mM perchlorate (Table 1, initial run). All results obtained were compared to untreated triplicate control chemostats. Upon treatment, sulfide decreased rapidly from 2.0 ± 0.57 mM and stabilized at 0.17 ± 0.076 mM (Figure 1A and Supplementary Figure S4A). This decrease in sulfide corresponded to a decline in the SRM population, as evidenced by qPCR of the *dsrA* gene as well as by the percentage of 16S rRNA amplicons assigned to known SRM (Figure 1B,C). SRM represented 33 ± 8.2% of the steady-state community, and fell to 1.3 ± 0.33% during treatment. Rebound post-treatment was immediate, and pre-treatment levels of sulfide were achieved within ∼200 h (4.2 resident times).

**FIGURE 2:**
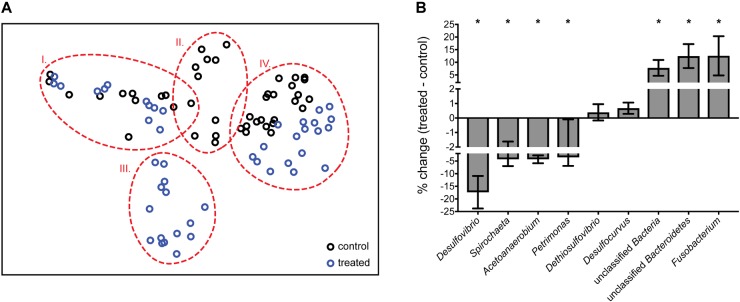
**(A)** nMDS (non-metric multi-dimensional scaling) plot of normalized and transformed 16S amplicon community profiles for control (black) and treated (blue) communities. Dashed red lines group samples at 70% similarity. Stress value of 0.1. Group I: pre-treatment; groups II and III: treatment; group IV: post-treatment **(B)** % change between control and treated samples during treatment (**A**, group II vs. III) of genera identified as important by SIMPER analysis. Error bars represent SD. ^∗^Genera which differ significantly via ANOVA (*p* < 0.001) between control and treated communities.

The 16S rRNA amplicon data generated allowed us to dissect community-wide effects of perchlorate. An nMDS plot shows that changes in communities cluster by treatment as well as by time (groups I–IV), with treated communities undergoing the same temporal effect as control communities, and rebounding to the same end-point. Control and treated communities group together before and after the treatment phase (at > 70% similarity), and separate, at 35% dissimilarity, during treatment (Figure 2A). SIMPER analysis reveals that 6 groups contribute over 50% to the total difference between treated and control communities during treatment: *Desulfovibrio*, *Spirochaeta*, *Acetoanaerobium*, *Petrimonas*, *Fusobacterium*, and a group of unclassified *Bacteroidetes* and Bacteria. *Desulfovibrio* decline from 19 ± 6.7% to <1% of the community, representing the largest contribution (11%) to the difference between treated and control communities during treatment. Several non-SR organisms also decrease significantly with treatment: *Spirochaeta*, *Acetoanaerobium*, and *Petrimonas* (Kruskall–Wallis test, *p* < 0.001). Conversely, during treatment, there is a relative enrichment of other, likely fermentative organisms, including Fusobacterium, the unclassified Bacteroidetes, and other unclassified Bacteria. Interestingly, *Desulfocurvus*, the only other SRM present in any significant proportion, as well as *Dethiosulfovibrio* were unaffected by perchlorate treatment (Figure 2B).

**FIGURE 3:**
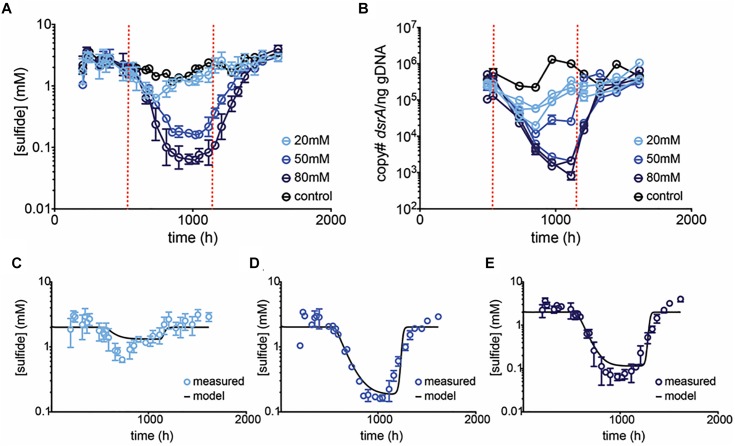
Average **(A)** sulfide production, and **(B)** *dsrA* copy number per ng gDNA in control (black circles) and treated (blue circles) chemostats. Dashed red lines indicate start and stop of treatment. Error bars represent SD from 3 replicates for 20 and 80 mM treatments and the range of 2 replicates for 50 mM treatment. In **(B)** replicates are plotted independently. Model prediction (black line) and empirical data (blue circles) of [sulfide] over time at **(C)** 20 mM, **(D)** 50 mM, and **(E)** 80 mM perchlorate are compared. Model assumes a *K*_p_ of 40%, a K_A_ of 0.5%, and μ of 0.13.

A subsequent chemostat experiment was performed using 20, 50, and 80 mM perchlorate (Table 1, validation run). At 50 mM perchlorate, very similar behavior was observed to that in the initial chemostat experiment, with sulfide dropping from 2.2 ± 0.64 mM to 0.16 ± 0.010 mM (Figure 3 and Supplementary Figure S4B). At 80 mM perchlorate, inhibition was the most pronounced (decreased to 0.067 ± 0.022 mM). Once again, rapid rebound was observed upon cessation of treatment (Supplementary Figure S5). The qPCR data validates these trends. The inhibitory effect of 20 mM perchlorate is less clear. At 20 mM, the system seems significantly inhibited initially, with sulfide concentrations falling to 0.64 ± 0.060 mM (*p* = 0.0012, *t*-test for 20 mM 660–852 h vs. all control at all time-points), but then rebounding to control levels within 250 h (*p* = 0.2 for 20 mM at 899–1114 h vs. control at all time-points). qPCR of *dsrA* shows a trend of slight inhibition of SRM throughout treatment, although this trend is non-significant (*p* = 0.4 for 20 mM 539–1114 h vs. control at all time-points). In subsequent runs, there was no significant effect of 20 mM perchlorate on sulfide production.

### Model Parameterization and Validation

A theoretical model of inhibition was constructed based on the assumption that perchlorate affects the growth rate of SRM. In support of this, it has been shown that SRM can overcome perchlorate inhibition via de-repression of the SR pathway (i.e., perchlorate is not biocidal but rather competes with sulfate for enzyme active sites), and additionally that pyruvate-fermenting *D. alaskensis* G20 cells are uninhibited by >100 mM perchlorate (Carlson et al., 2015a,b). To confirm this non-biocidal effect, triplicate populations of *Desulfovibrio* sp. BMSR were exposed to 50 mM perchlorate for 24 h, with and without lactate (donor). The proportion of dead:live cells was compared to that in control cultures with no lactate and no perchlorate, via live-dead staining. All cultures exhibited a similar proportion of dead cells post-incubation (21 ± 6.0%, *p* > 0.2 for all pairwise comparisons via Kruskal–Wallis test).

The max growth rate (μ_max_ = 0.13*h*^−1^) for the model was determined from batch growth curves of community sulfide production. The dose-response/inhibition curve of perchlorate against SRM was also determined empirically. The final function for quantifying the perchlorate effect (*y*) on μ is based on growth rates (Supplementary Figures S2A,B), and is as follows:

(7) $$y=\frac{1}{1+10^{\left(-1,914-\log[P])\;(-1.813\right)}}$$

From here, *y* is multiplied by μ_max_ (0.13*h*^−1^) to give the actual growth rate, μ. In the final model, the population of SRM then follows Equations 4a – 4c.

The model was calibrated using data from the initial chemostat run. *N_t_* was set to 40% based on the average % SRM in control chemostats over time (∼ steady state SRM population). Several surface-attached fractions were simulated before settling on*N_A_* = 0.5%. The model output was converted from % SRM to [sulfide], according to the regression of log(sulfide) against log(%reads) (*R*^2^ = 0.79):

(8) $$[\mathrm{sulfide}]=10^{\left(0.6786\times \log(N_{t})-0.7375\right)}$$

The model was then validated against the sulfide data generated in the subsequent validation chemostat run at 20, 50, and 80 mM perchlorate (Figure 3C–E). Predictions agree well with empirical results, supporting the model that perchlorate acts largely to decrease μ, rendering planktonic SRM increasingly susceptible to wash-out at constant hydraulic residence time. Simulation results highlighted the importance of several parameters: (1) The max growth rate, μ_max_ is critical in establishing rebound: if it is slower than the residence time, SRM do not rebound (Supplementary Figure S3A). (2) As the dose-response/inhibition curve is responsible for setting the actual μ, it also defines the shape of the inhibition curve (Supplementary Figure S3B). (3) Finally, surface attachment plays a role (Supplementary Figure S3C): in the absence of surface-attached SRM, sulfide decreases continuously, asymptotically toward 0. This is in contrast to empirical observations in the planktonic chemostats, where a steady “inhibited” sulfide level and SRM population were reached, which probably suggests that some surface-attachment is occurring on the vessel walls preventing washout.

### The Role of Surface-Attachment

**FIGURE 4:**
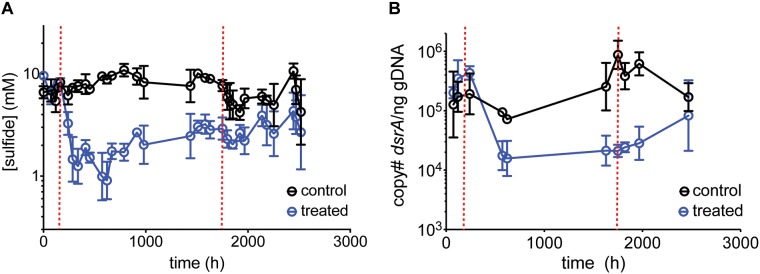
Average **(A)** sulfide production, **(B)** *dsrA* copy number per ng gDNA over time for control chemostats (black circles) and chemostats treated with 50 mM perchlorate (blue circles). Dashed red lines indicate start and stop of treatment. Error bars represent SD from 3 replicates.

Surface-attachment may alter inhibitory dynamics by preventing SRM washout. If surface-attached SRM constrain inhibition, then increasing their proportion relative to planktonic SRM should decrease the effectiveness of perchlorate and result in higher “inhibited” sulfide levels. To achieve this effect, we added glass beads to chemostats (biofilm culture chemostats), increasing surface area for attachment, and treated with 50 mM perchlorate (Table 1, surface-attachment run). This mode of operation resulted in higher levels of sulfide than previous experiments (7.5 mM ± 2.2 mM) performed with the planktonic culture chemostats. However, inhibition of sulfidogenesis by 50 mM perchlorate followed a similar pattern, and qPCR data validated this trend (Figure 4 and Supplementary Figure S4C). Normalized to the total sulfide production, the rate of inhibition (decline in sulfide production upon treatment) was not significantly different between planktonic and biofilm chemostats (Graphpad Prism linear regression analysis, *p* = 0.3). However, rebound in the biofilm culture chemostats was not as obvious as previously observed (Supplementary Figure S5).

**FIGURE 5:**
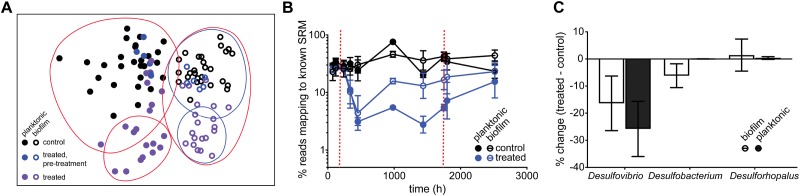
**(A)** nMDS plot of normalized and transformed 16S amplicon community profiles for control (black) and treated (purple: pre-treatment; blue: treated) communities. Red and blue lines group samples at 64 and 68% similarity, respectively. Stress value of 0.2. **(B)** Percentage of reads mapping to known SRM, based on 16S amplicon sequencing, over time for control chemostats (black circles; open = biofilm, closed = planktonic) and chemostats treated with 50 mM perchlorate (blue circles, open = biofilm, closed = planktonic). Dashed red lines indicate start and stop of treatment. **(C)** % change of dominant SRM between control and treated samples during treatment. Error bars represent SD.

Amplicon sequencing of planktonic and surface-attached populations revealed a community structure dominated by fermentative and sulfate-respiring organisms in both instances. Anaerobic biofilms are often stratified, with more favorable electron-accepting processes closer to the surface (Sun et al., 2014; Martin et al., 2015); in our system, a lack of alternative electron-accepting processes likely resulted in a heterogeneous, mixed structure. Sequencing data confirmed the inhibition of SRM. SIMPER analysis identified *Desulfovibrio* species as contributing 11% to the difference between control and treated planktonic communities, and 8% for biofilm communities. A nMDS plot shows control samples and pre-treatment samples group together, and the treated samples clustered independently. The same trend was true for biofilm communities. However, planktonic communities showed a greater separation than biofilm communities upon treatment (36% vs. 32% dissimilarity between control/pre-treatment and treatment groups) (Figure 5A). Examining the SRM population specifically confirmed that planktonic SRM were significantly more inhibited than surface-attached SRM (*p* = 0.011, Mann–Whitney test). Planktonic SRM dropped to 3.9 ± 1.4% of the community while surface-attached SRM dropped to 12 ± 6.5% (Figure 5B). The proportion of SRM in planktonic and surface-attached control communities, on the other hand, was not significantly different (*p* = 0.45, Mann–Whitney test). Interestingly, *Desulfobacterium* and *Desulforhopalus* were enriched in the surface attached SRM fraction as compared to the planktonic (Figure 5C). *Desulfobacterium* was inhibited during treatment, whereas *Desulforhopalus* remained unaffected.

### Indirect Effects of Perchlorate

While our experimental data and model confirm the direct inhibitory effect of perchlorate on SRM, particularly at concentrations greater than or equal to media sulfate concentrations (20 mM), perchlorate also has indirect effects on sulfate-reduction. Dissimilatory perchlorate reducing bacteria (DPRB) can reduce perchlorate coupled to the oxidation of organic acids, an energetically favorable metabolism compared to sulfate-reduction. Thus, DPRB can outcompete SRM for organic donors. Further, DPRB can metabolize sulfide, enzymatically oxidizing it to elemental insoluble sulfur (Engelbrektson A. et al., 2014; Gregoire et al., 2014; Cheng et al., 2016; Mehta-Kolte et al., 2017). Previous work has been unable to empirically distinguish between direct and indirect effects, although modeling of packed columns studies has indicated that indirect effects are predominant in controlling sulfide production under steady state conditions (Engelbrektson A.L. et al., 2018). Since the community in this study did not contain DPRB, it presented us with an opportunity to more specifically test the magnitude of indirect effects by amending treated communities with DPRB.

**FIGURE 6:**
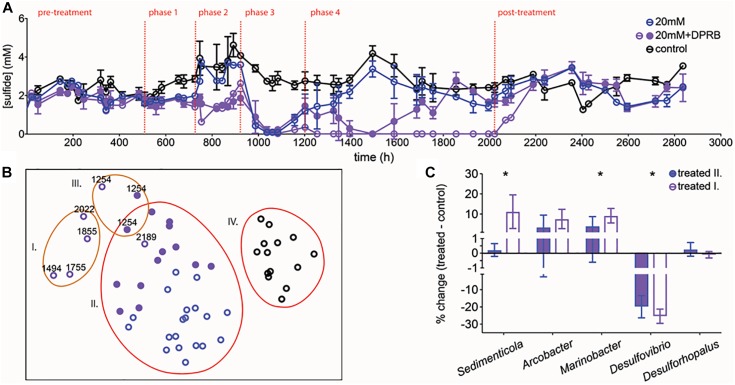
**(A)** Average sulfide production in control chemostats (black circles), chemostats with 20 mM perchlorate (blue circles) and chemostats with 20 mM perchlorate, amended with DPRB (purple circles). Amended chemostats did not replicate each other and hence the two trajectories are shown as closed circles (replicates 1 and 2) and open circles (replicate 3). Dashed lines indicate start/stop of various treatments (refer to main text). Error bars represent SD for replicates. **(B)** nMDS plot of normalized and transformed 16S amplicon community profiles for control (black circles) and treated (20 mM: blue circles, 20 mM+DPRB: purple circles; open circles represent replicate 3) communities, during phase 4. Orange and red lines group samples at 65 and 70% similarity, respectively. Stress value of 0.2. Numbers next to symbols represent time (h) for sample. **(C)** % change in DPRB and SRM between groupings I or II and the control group IV. Grouping I represents samples from replicate 3 (the inhibited 20 mM + DPRB chemostat); grouping II represents samples from 20 mM perchlorate chemostats and replicates 1 and 2 of the 20 mM + DPRB chemostats. Groupings were defined based on % similarity as outlined in the nMDS plot (orange and red circles in **B**). Error bars represent SD. ^∗^Genera which differ significantly via ANOVA (*p* < 0.01) between groupings I. and II.

Amendment with a mix of DPRB (1:10 inoculum) was initially tested in batch communities treated with 20 mM perchlorate. This perchlorate concentration was chosen as it is minimally directly inhibitory to sulfidogenesis in our system, and thus allows us to maximally detect indirect inhibitory effects. Treatment with 20 mM perchlorate led to complete sulfide removal coupled to the reduction of 7.7 ± 2.6 mM perchlorate. Indirect effects of perchlorate were subsequently tested in chemostats (Table 1, DPRB run). Planktonic sulfidogenic communities were allowed to reach steady sulfide production and amended with either 20 mM perchlorate or 20 mM perchlorate and a spike of DPRB (phase 1). The initial amendment with DPRB had no significant effect on sulfide production (Figure 6A). The continuous generation of sulfide may have forced DPRB to continuously oxidize it, which they cannot couple to growth (Mehta-Kolte et al., 2017), thus causing them to be washed out of the system. To prevent washout, 60 mM lactate alone or with the DPRB was spiked into chemostats and flow was shut off (phase 2). In this treatment phase, sulfide production was enhanced in both the control and 20 mM treated chemostats, but remained level in 20 mM + DPRB chemostats. Thus, the DPRB did indeed inhibit sulfide production, but were not able to completely eliminate sulfide. In phase 3, flow was reinstated and perchlorate was replaced with 20 mM nitrate, in order to give perchlorate reducers, most of whom preferentially reduce nitrate, a competitive advantage (Chaudhuri et al., 2002; Coates and Achenbach, 2004). This completely eliminated sulfide in all chemostats containing nitrate, irrespective of the presence of DPRB. Nitrate reduction and nitrite production was observed in both amended and un-amended communities (Supplementary Figure S4D), likely due to native organisms capable of nitrate reduction, such as *Shewanella*. Nitrate treatment was suspended and perchlorate (20 mM) or perchlorate and a large inoculum (30%) of DPRB was added (phase 4). This eliminated sulfide in the perchlorate and DRPB-amended replicates, briefly. However, subsequent to and likely as a result of issues with flow, 20 mM and 20 mM + DPRB treatments became indistinguishable again in two out of the three replicates, starting at hour 1700. One replicate (replicate 3) did maintain complete sulfide inhibition for the duration of perchlorate treatment, and concurrent perchlorate reduction was seen (Supplementary Figure S4D). Interestingly, this chemostat displayed the lowest sulfide level at the start of phase 4, which likely helped in the establishment of DPRB.

An nMDS of the microbial community data during phase 4 confirmed that the community in replicate 3 was different when compared to replicates 1–2 and 20 mM perchlorate-only chemostats (group I vs. group II, respectively, Figure 6B). Comparing groups I and II revealed a significantly greater presence of *Sedimenticola* in group I, as well as a greater enrichment of *Marinobacter* (Figure 6C). The inhibition of SRM *Desulfovibrio* and *Desulfohorpalus* was also enhanced in group I compared to group II. Finally, the significant inhibition of a group of unclassified Bacteria in I compared to II suggested a possible negative interaction between these microbes and the DPRB.

### Inhibitory Dynamics in *Desulfovibrio* sp. BMSR

To further understand direct perchlorate inhibition of SRM in the chemostats, the dominant SRM from this community, *Desulfovibrio* sp. strain BMSR (IMG genome ID 2684623040), was isolated and tested in an additional chemostat run with 20 mM or 50 mM perchlorate (Table 1, pure-culture run). The isolate, *Desulfovibrio* sp. BMSR, was characterized in terms of max growth rate and dose-response/inhibition for perchlorate (Supplementary Figures S2C,D).

$${\text{μ}}_{\max}=0.2$$

$$y=\frac{1}{1+10^{\left(-2.099-\log[P])\;(-1.108\right)}}$$

**FIGURE 7:**
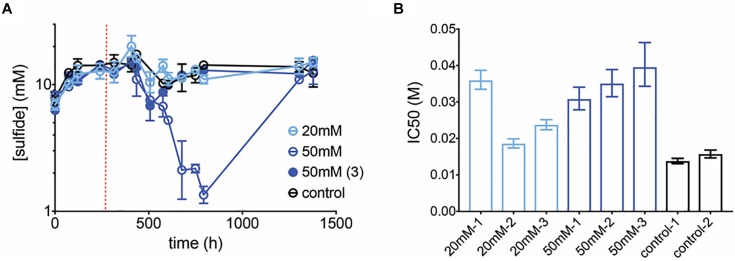
**(A)** Average sulfide production in control (black circles) and treated (blue circles) pure-culture *D.* sp. BMSR chemostats. Chemostats treated at 50 mM did not replicate each other and hence both trajectories are shown (replicates 1–2: open circles, replicate 3: closed circle). Dashed line indicates start of treatment. Error bars represent SD of replicates. **(B)** IC50 values for all chemostats, measured at final time-point. Error bars represent the 95% CI of the IC50 estimate.

The max growth rate is higher than for the community but the inhibition curve is similar (Supplementary Figure S2). Perchlorate at 20 mM showed no significant effect on sulfide production (*p* = 0.8 *t*-test for control vs. 20 mM treatments over all replicates and treated time-points). However, at 50 mM treatment, sulfide concentrations demonstrated a pronounced decline in two out of three replicates (Figure 7A). Sulfide measurements for the 3rd replicate dropped briefly, but recovered and were unaffected thereafter. In contrast to earlier community experiments, sulfide inhibition lagged, i.e., sulfide was unaffected for over 100 h after treatment was initiated. Further, in this pure culture experiment, two of the inhibited replicates rebounded to control sulfide levels although perchlorate amendment remained constant. The communities were tested in batch for their susceptibility to perchlorate, and indeed revealed adaptation to the inhibitor, with IC50s for all treated cultures increasing 1.2 to 2.6-fold over controls (Figure 7B).

Several additional, as of yet unexplored, factors may affect sensitivity of SRM to perchlorate. First, the electron donor for sulfate-reduction in our community was unknown and was likely a complex mixture as the community experiments were performed on yeast extract. Hence, the effect of donor on IC50 was elucidated using *Desulfovibrio* sp. BMSR grown on a variety of carbon sources (lactate, pyruvate, fumarate, succinate, glucose) and 20 mM sulfate. Interestingly, perchlorate sensitivity was donor dependent, particularly in the case of pyruvate (*p* < 0.0001, Extra sum-of-squares *F* test) (Supplementary Figure S6). Furthermore, the community contained a mixed SRM population in which each individual SRM likely displayed a different sensitivity to perchlorate. The hypothesis that the sensitivity of the mixed SRM culture is determined by the sensitivity of the most resistant SRM was tested by co-culturing *Desulfovibrio* sp. BMSR with *D. alaskensis* G20. G20 is more resistant to perchlorate and, as expected, the co-culture (consisting of the two isolates in a 1:1 initial ratio) had the same dose-response curve against perchlorate as the pure-culture G20 (Supplementary Figure S7A). Finally, in addition to the SR fraction, our model community contains a fermentative fraction, likely involved in the degradation of components of yeast extract not utilizable by SRM. The presence of fermentative organisms might be either antagonistic or synergistic with perchlorate inhibition. In order to address this, the fermentative fraction of the community was sub-cultured in media lacking sulfate and subsequently combined with *Desulfovibrio* sp. BMSR at a 1:1 ratio. Here again, sensitivity to perchlorate of the fermenter-amended co-culture was dictated by the SRM (Supplementary Figure S7B). Thus, strikingly, the inhibitory response of a mixed community growing on a specific electron donor may be modeled based on the sensitivity of the most-resistant SRM in that community, and on that particular donor.

## Discussion

The aim of this study is to elucidate the various mechanisms of perchlorate inhibition in complex sulfidogenic communities. The initial chemostat experiment confirms that perchlorate at 50 mM is a potent direct inhibitor of SRM. SRM inhibition coincides with the relative enrichment of several presumably fermentative organisms. Members of the *Fusobacterium* genus are known fermenters, producing butyrate and acetate from various sugars, amino acids and small peptides and the enrichment of *Fusobacterium* under perchlorate treatment has been previously characterized (Kapatral et al., 2005; Engelbrektson A. et al., 2014). The same ecological niche likely exists for the unclassified *Bacteroidetes* and Bacteria comparatively enriched during treatment. Several non-SRM were negatively affected by treatment. Members of *Spirochaeta* are typically fermentative but some species require sulfide (Leschine et al., 2006; Dubinina et al., 2011). Similarly, the growth of *Petrimonas*, a presumed fermenter, can be stimulated by the presence of sulfur (Grabowski et al., 2005) and a decline in sulfide may have led to a decline in abiotically produced sulfur. Interestingly, the SRM *Dethiosulfovibrio* and *Desulfocurvus* were not significantly affected by the presence of perchlorate; these genera may be persisting via thiosulfate reduction (Magot et al., 1997; Surkov et al., 2001; Takii et al., 2007; Klouche et al., 2009; Hamdi et al., 2013) produced as a result of sulfur redox cycling as proposed previously (Engelbrektson A. et al., 2014). Patterns of inhibition and relative enrichment are similar across all chemostat runs and indicate that the largest effect is on the dominant SRMs, *Desulfovibrio* spp.

By combining empirical data with mathematical modeling, we were able to further our understanding of perchlorate inhibition. Models of microbial growth often include a substrate parameter, as substrate concentration and type govern microbial growth rates (Kovarova-Kovar and Egli, 1998; Rittman and McCarty, 2001; Meyer et al., 2013). In this study, the donor for SR remains unknown and is likely multivariate. To exclude this parameter from the model, μ was empirically determined in batch, on yeast extract and sulfate, and assumed to be relevant to chemostats operated on the same electron donor and acceptor. Further, the lack of known electron donor and specific nutrient requirements for SR in our complex system necessitates an alternative mechanism to limit the SRM population. A “carrying capacity” term, *K*, was chosen and empirically determined. In this way, the complexity of the system is reduced to two parameters: the empirical μ of the mixture of SRM on a mixture of donors, and the empirical *K*, the maximum sustainable SRM population size in this system. Both parameters are assumed to stay constant over the course of inhibition. μ is a critical model parameter as it encompasses the effect of perchlorate inhibition and determines both the level of inhibition as well as the ability of sulfidogenesis to rebound post-treatment. In a system where flow is faster than μ, no rebound can occur. The second critical parameter is the dose-response/inhibition curve itself, which we empirically determined via batch growth curves. The defined function accurately captured the inhibition profile in chemostats, confirming both that inhibition in batch can be used to predict inhibition in continuous-flow systems and that, in continuous-flow, the total effect of inhibition can be constrained to the effect on μ, the growth rate. The model is predictive at 50 and 80 mM perchlorate. Interestingly, 80 mM perchlorate does not seem to dramatically increase inhibition relative to 50 mM, suggesting a threshold concentration, above which treatment is not economical. At 20 mM, there is a slight decoupling between the effect on sulfide and the effect on *dsrA* copy number. *Desulfovibrio alaskensis* G20, when exposed to competitive inhibitors, can upregulate the pathway for SR and slightly decrease its sensitivity to perchlorate (Carlson et al., 2015a). While this slight advantage would be less relevant at high perchlorate concentrations, at low concentrations it may overcome the inhibitory effect on sulfide production. Furthermore, several genera of non-SRM in this system have the capacity for low-level sulfide production upon cysteine or methionine degradation, or directly from elemental sulfur (*Fusobacterium*, *Spirochaeta*) (Kapatral et al., 2005; Leschine et al., 2006). At low concentrations of perchlorate, these alternative mechanisms might represent a larger confounding source of sulfide.

The model also emphasizes the importance of surface attachment. SRM in biofilms are less subject to wash-out and hence can maintain a certain low level of sulfide production. Surface area was experimentally manipulated via the addition of glass beads. Although these chemostats were not stirred, diffusion did not seem to be a major issue (likely due to mixing during daily sampling events) as perchlorate concentrations followed the uniformly mixed model, which would not be expected in a non-uniform, diffusion-limited system (Supplementary Figure 5C). It is also important to note that surface-attached and planktonic populations were modeled independently; in reality they are likely interconverting and interdependent. This is a complication that is not relevant to model predictions and functioning, as both populations are assumed to be affected by perchlorate according to the same function. It is possible that biofilm cells are innately more resistant to competitive inhibition (different inhibition function). The alignment of inhibition rates between planktonic and biofilm cultures, however, suggests otherwise. Furthermore, current evidence for overcoming perchlorate inhibition is limited to de-repression of the *sat* gene (Carlson et al., 2015a), which should be a similar effect in biofilm and planktonic cultures. There is currently no evidence suggesting that SRM upregulate central genes for sulfate-respiration as a specific response to growth in biofilms (Clark et al., 2012). As noted previously, the biofilm chemostats produced more sulfide, perhaps due to a change in the relative proportions of the dominant sulfate-reducers. In any case, the level of inhibition was more limited, with sulfide stabilizing at a higher level, in keeping with a higher level of surface attachment. The relative persistence of surface-attached populations was verified by amplicon sequencing of the biofilm and planktonic community fraction. Previous studies in our lab also show that SRM can persist in sediment flow-through systems upon treatment (Engelbrektson A.L. et al., 2018), possibly a combination of surface-attachment and SRM performing fermentative metabolisms which are largely unaffected by perchlorate. Thiosulfate reduction may also present an alternative mechanism for persistence. Indeed, *Dethiosulfovibrio* are consistently an important part of perchlorate-treated communities.

We also explored indirect effects of perchlorate inhibition. At concentrations of perchlorate that are minimally directly inhibitory (≈20 mM), DPRM can eliminate sulfide completely. However, in our chemostats, the establishment of DPRM and extent of inhibition was dependent on the community context and the existing sulfide concentrations. Perchlorate treatment in the field would enrich for native organisms capable of perchlorate reduction, likely more competitive in their given environment than exogenously introduced DPRM. Previous work in our lab has successfully enriched for perchlorate reduction in marine systems, concomitant with total suppression of sulfide (Engelbrektson A. et al., 2014, 2018; Cheng et al., 2016; Engelbrektson A.L. et al., 2018). These were sediment systems and spatial stratification likely played a role in helping enrich for perchlorate reducers: DPRM could be spatially separated from SRM and from sulfide, whereas in our chemostats DPRM were constantly encountering high levels of sulfide which can be inhibitory to their growth (Mehta-Kolte et al., 2017). Interestingly, in the chemostats, nitrate treatment had a drastic effect on sulfide production. Several genera (*Shewanella*, *Denitrovibrio*, *Petrimonas*) are capable of nitrate reduction and nitrite accumulation. Nitrite is a known potent inhibitor of SRM and a current mechanism for reversing sulfide production in commercial oil reservoirs, which has been applied with varying degrees of success in various systems (Nemati et al., 2001; Greene et al., 2003; Lambo et al., 2008; Voordouw et al., 2009; Callbeck et al., 2011, 2013; Gieg et al., 2011). Our results, alongside previous studies, suggest that simultaneous treatment with nitrate and perchlorate may represent a promising strategy, suppressing sulfidogenesis at low concentrations of these oxyanions and minimizing their environmental burden (Engelbrektson A. et al., 2018).

Inhibition may also be modulated by donor used for growth. In our system, *Desulfovibrio* sp. BMSR grown on pyruvate-sulfate is significantly more resistant than when grown on lactate-sulfate. The energetics and electron flow of pyruvate-sulfate grown *D. alaskensis* G20 and lactate-sulfate grown G20 are known to differ: electrons from lactate can flow through H_2_ as an electron carrier, whereas electrons from pyruvate can flow directly and rapidly to SR through the menaquinone pool (Keller et al., 2014; Zhou et al., 2017). Currently, the known mechanism for perchlorate resistance is overexpression of *sat*; Sat is regulated by the redox-sensitive repressor, Rex (Carlson et al., 2015a; Christensen et al., 2015). It is possible that the donor used for growth affects Rex directly, or more generally affects the redox state of the cell and causes changes in the expression of *sat*. There is some evidence to suggest that *sat* expression changes with pyruvate vs. lactate as the donor, though it is inconclusive (Keller and Wall, 2011)^1^. It is also possible that sulfate transport (and thereby perchlorate transport) into cells is partially donor dependent. How these various factors translate to donor-dependent differences in sensitivity remains to be addressed. Further, sensitivity of BMSR did not depend on whether it was grown in pure culture or in co-culture with other fermenters. As such, at least in this system, inhibition of SRM is independent of other community members. The *Desulfovibrio* sp. BMSR chemostat run with 20 and 50 mM perchlorate mimics community results, and can be modeled by adjusting μ, the interconversion factor between *N_t_* and [*sulfide*] and the dose-response/inhibition curve of perchlorate. Hence, remarkably, whether in a complex community or in a pure culture, the batch determination of a few parameters can be used to predict inhibition. Finally, this pure-culture chemostat study uncovered the relative ease with which pure cultures of SRM adapt to perchlorate. Adaptation of SRM in the community was not seen over the course of this study, although it was specifically assayed for after each chemostat run. Adaptation has also not been seen in other long-term community studies of perchlorate inhibition in this lab, making our observation of adaption in pure-cultures all the more interesting. Future work will aim to describe mechanisms of adaptation to perchlorate, both in pure and mixed culture.

In summary, the addition of perchlorate leads to slower growth of SRM populations, which renders them susceptible to washout. This effect can be partially mitigated by increased surface-attachment. This principal governs inhibition in a pure culture as well as in a mixed community. The data presented here highlight how simple mathematical models constrained by a few experimentally determined parameters in batch can predict the behavior of complex continuous systems. Isolation, combined with the creation of synthetic co-cultures and mixed communities, allows us to parse out indirect effects of perchlorate as well as to define controls on inhibition when multiple organisms are present. In this way, this work provides both a framework for testing community-relevant ecological and physiological hypotheses, as well as practical insights for controlling sulfidogenesis across environments.

## Data Availability

The datasets generated for this study can be found in NCBI, PRJNA521657.

## Author Contributions

MS and JC designed the study. MS performed the experiments with the help of GN and NG. YC helped with modeling. MS wrote manuscript, with edits from AE, HC, and JC.

## Conflict of Interest Statement

JC holds IP on the application of perchlorate to oil reservoir souring. The remaining authors declare that the research was conducted in the absence of any commercial or financial relationships that could be construed as a potential conflict of interest.

## Footnotes

1 http://www.microbesonline.org/cgi-bin/microarray/geneExpress.cgi?locusId=206736&shrunk=n&expType=RNA

## Supplementary Material

FIGURE S1 **(A)** Chemostat design: anaerobic media (influent) is pumped in; effluent (waste culture) drains passively. A culture of approximately 125 ml is maintained and sampled through the sampling port. **(B)** Experimental design: communities are allowed to reach stable sulfide production before treatment with perchlorate (t_START_). Various chemical parameters are monitored during inhibition and once inhibition levels off, treatment is stopped (t_STOP_) and rebound is monitored.

FIGURE S2 Growth curves of **(A)** community sulfide production and **(C)** *Desulfovibrio* sp. BMSR, at various perchlorate concentrations. Perchlorate was spiked in at 12 and 7 h respectively. Data generated was used to construct dose-response/inhibition curves for **(B)** community SRM and **(D)** *D.* sp. BMSR. Error bars represent SD of three replicates.

FIGURE S3 Model predictions for the effect of **(A)** increasing μ, **(B)** changing the dose-response/inhibition curve (IC50/Hillslope parameters) and **(C)** increasing *N_t_*, while keeping all other model parameters constant.

FIGURE S4 Perchlorate concentrations (circles– measured, solid line – modeled) for **(A)** initial chemostat run at 50 mM, **(B)** subsequent run at 20, 50, and 80 mM, and **(C)** surface-attachment run at 50 mM. **(D)** Measured perchlorate concentrations in DPRB-amendment run, for chemostats treated with 20 mM perchlorate (blue symbols), and 20 mM perchlorate and DPRB (purple symbols). The latter treatment is split into replicates 1–2 (open symbols) and replicate 3 (closed symbols). Nitrate and nitrite concentrations are also given, for the brief period of nitrate treatment, indicated by dashed red lines. Error bars represent SD.

FIGURE S5 Slope of the linear regression fit to log transformed sulfide data during the period of inhibition and the period of rebound for each respective chemostat experiment. Bars represent the 95% CI.

FIGURE S6 IC50 measurements for *Desulfovibrio* sp. BMSR on different donors: lac, sodium lactate; pyr, sodium pyruvate; fum, sodium fumarate; suc, sodium succinate; and glu, glucose. Error bars represent the 95% CI of the IC50 estimate.

FIGURE S7 Dose-response/inhibition curves for **(A)** *Desulfovibrio alaskensis* G20 (black circles), *Desulfovibrio* sp. BMSR (light gray circles) and a co-culture of both organisms (gray circles) and **(B)** *Desulfovibrio* sp. BMSR in pure culture (black circles) and co-cultured with a mixed community of fermenters (gray circles). Error bars represent the SD of four replicates.

## References

[B1] Barton L. L. Fauque G. D. (2009). “Biochemistry, physiology and biotechnology of sulfate-reducing bacteria,” in *Advances in Applied Microbiology*, ed. Laskin A. (Amsterdam: Elsevier Inc.),41–98. 10.1016/S0065-2164(09)01202-7 19426853

[B2] Bourne D. G. Muirhead A. Sato Y. (2011). Changes in sulfate-reducing bacterial populations during the onset of black band disease. *ISME J.* 5 559–564. 10.1038/ismej.2010.143 20811471 PMC3105726

[B3] Bruce R. A. Achenbach L. A. Coates J. D. (1999). Reduction of (per)chlorate by a novel organism isolated from paper mill waste. *Environ. Microbiol.* 1 319–329. 10.1046/j.1462-2920.1999.00042.x 11207750

[B4] Callbeck C. M. Dong X. Chatterjee I. Agrawal A. Caffrey S. M. Sensen C. W. (2011). Microbial community succession in a bioreactor modeling a souring low-temperature oil reservoir subjected to nitrate injection. *Appl. Microbiol. Biotechnol.* 91 799–810. 10.1007/s00253-011-3287-2 21538114

[B5] Callbeck C. M. C. Agrawal A. Voordouw G. (2013). Acetate production from oil under sulfate-reducing conditions in bioreactors injected with sulfate and nitrate. *Appl. Environ. Microbiol.* 79 5059–5068. 10.1128/AEM.01251-13 23770914 PMC3754712

[B6] Carlson H. K. Kuehl J. V. Hazra A. B. Justice N. B. Stoeva M. K. Sczesnak A. (2015a). Mechanisms of direct inhibition of the respiratory sulfate-reduction pathway by (per)chlorate and nitrate. *ISME J.* 9 1295–1305. 10.1038/ismej.2014.216 25405978 PMC4438318

[B7] Carlson H. K. Stoeva M. K. Justice N. B. Sczesnak A. Mullan M. R. Mosqueda L. A. (2015b). Monofluorophosphate is a selective inhibitor of respiratory sulfate-reducing microorganisms. *Environ. Sci. Technol.* 49 3727–3736. 10.1021/es505843z 25698072

[B8] Carlström C. I. Loutey D. E. Wang O. Engelbrektson A. Clark I. Lucas L. N. (2015). *Selenatireducens* strain CUZ, a marine ( Per ) chlorate-respiring. *Appl. Environ. Microbiol.* 81 2717–2726. 10.1128/AEM.03606-14 25662971 PMC4375318

[B9] Carlström C. I. Lucas L. N. Rohde R. A. Haratian A. Engelbrektson A. L. Coates J. D. (2016). Characterization of an anaerobic marine microbial community exposed to combined fluxes of perchlorate and salinity. *Appl. Microbiol. Biotechnol.* 100 9719–9732. 10.1007/s00253-016-7780-5 27596621

[B10] Carlström C. I. Wang O. Melnyk R. A. Bauer S. Lee J. Engelbrektson A. (2013). Physiological and genetic description of dissimilatory perchlorate reduction by the novel marine bacterium *Arcobacter* sp. *Strain CAB. MBio* 4 1–9. 10.1128/mBio.00217-13 23695836 PMC3656443

[B11] Chaudhuri S. K. Connor S. M. O. Gustavson R. L. Achenbach L. A. Coates J. D. (2002). Environmental factors that control microbial perchlorate reduction. *Appl Environ Microbiol.* 68 4425–4430. 10.1128/AEM.68.9.4425 12200296 PMC124121

[B12] Cheng Y. Hubbard C. G. Li L. Bouskill N. Molins S. Zheng L. (2016). Reactive transport model of sulfur cycling as impacted by perchlorate and nitrate treatments. *Environ. Sci. Technol.* 50 7010–7018. 10.1021/acs.est.6b00081 27267666

[B13] Christensen G. A. Zane G. M. Kazakov A. E. Li X. Rodionov D. A. Novichkov P. S. (2015). Rex (Encoded by DVU_0916) in *Desulfovibrio vulgaris* Hildenborough is a repressor of sulfate adenylyl transferase and is regulated by NADH. *J. Bacteriol.* 197 29–39. 10.1128/JB.02083-14 25313388 PMC4288696

[B14] Clark M. E. He Z. Redding A. M. Joachimiak M. P. Keasling J. D. Zhou J. Z. (2012). Transcriptomic and proteomic analyses of *Desulfovibrio vulgaris* biofilms: carbon and energy flow contribute to the distinct biofilm growth state. *BMC Genomics* 13:138. 10.1186/1471-2164-13-138 22507456 PMC3431258

[B15] Clarke K. (1993). Non-parametric multivariate analyses of changes in community structure. *Aust. J. Ecol.* 18 117–143.

[B16] Clarke R. (2015). *PRIMER v7: User Manual/Tutorial*. Available at: https://www.coursehero.com/file/31450113/PRIMER7-UsersMethodsmanualv7pdf/

[B17] Cline J. (1969). Spectrophotometric determination of hydrogen sulfide in natural waters. *Limnol. Oceanogr.* 14 454–458. 10.4319/lo.1969.14.3.0454

[B18] Coates J. D. Achenbach L. A. (2004). Microbial perchlorate reduction?: rocket-fuelled metabolism. *Nat Rev Microbiol.* 2 569–580. 10.1038/nrmicro926 15197392

[B19] Dubinina G. Grabovich M. Leshcheva N. Rainey F. A. Gavrish E. (2011). Spirochaeta perfilievii sp. nov., an oxygen-tolerant, sulfide-oxidizing, sulfur- and thiosulfate-reducing spirochaete isolated from a saline spring. *Int. J. Syst. Evol. Microbiol.* 61 110–117. 10.1099/ijs.0.018333-0 20173011

[B20] Edgar R. C. Haas B. J. Clemente J. C. Quince C. Knight R. (2011). UCHIME improves sensitivity and speed of chimera detection. *Bioinformatics* 27 2194–2200. 10.1093/bioinformatics/btr381 21700674 PMC3150044

[B21] Engelbrektson A. Briseno V. Figueroa I. Yee M. Shao G. L. Carlson H. K. (2018). Mitigating sulfidogenesis with simultaneous perchlorate and nitrate treatments. *Front. Microbiol.* 9:2305. 10.3389/fmicb.2018.02305 30337913 PMC6180152

[B22] Engelbrektson A. L. Cheng Y. Hubbard C. G. Jin Y. T. Arora B. Tom L. M. (2018). Attenuating sulfidogenesis in a soured continuous flow column system with perchlorate treatment. *Front. Microbiol.* 9:1575. 10.3389/fmicb.2018.01575 30140256 PMC6094985

[B23] Engelbrektson A. Hubbard C. G. Tom L. M. Boussina A. Jin Y. T. Wong H. (2014). Inhibition of microbial sulfate reduction in a flow-through column system by (per)chlorate treatment. *Front. Microbiol.* 5:315. 10.3389/fmicb.2014.00315 25071731 PMC4092371

[B24] Enning D. Garrelfs J. (2014). Corrosion of iron by sulfate-reducing bacteria: new views of an old problem. *Appl. Environ. Microbiol.* 80 1226–1236. 10.1128/AEM.02848-13 24317078 PMC3911074

[B25] Gieg L. M. Jack T. R. Foght J. M. (2011). Biological souring and mitigation in oil reservoirs. *Appl. Microbiol. Biotechnol.* 92 263–282. 10.1007/s00253-011-3542-6 21858492

[B26] Grabowski A. Tindall B. J. Bardin V. Blanchet D. Jeanthon C. (2005). Petrimonas sulfuriphila gen. nov., sp. nov., a mesophilic fermentative bacterium isolated from a biodegraded oil reservoir. *Int. J. Syst. Evol. Microbiol.* 55 1113–1121. 10.1099/ijs.0.63426-0 15879242

[B27] Greene E. A. Hubert C. Nemati M. Jenneman G. E. Voordouw G. (2003). Nitrite reductase activity of sulphate-reducing bacteria prevents their inhibition by nitrate-reducing, sulphide-oxidizing bacteria. *Environ. Microbiol.* 5 607–617. 10.1046/j.1462-2920.2003.00446.x 12823193

[B28] Gregoire P. Engelbrektson A. Hubbard C. G. Metlagel Z. Csencsits R. Auer M. (2014). Control of sulfidogenesis through bio-oxidation of H2S coupled to (per)chlorate reduction. *Environ. Microbiol. Rep.* 6 558–564. 10.1111/1758-2229.12156 25756108

[B29] Hamdi O. Hania W. Ben Postec A. Bartoli M. Hamdi M. Bouallagui H. (2013). Isolation and characterization of *Desulfocurvus thunnarius* sp. nov., a sulfate-reducing bacterium isolated from an anaerobic sequencing batch reactor treating cooking wastewater. *Int. J. Syst. Evol. Microbiol.* 63 4237–4242. 10.1099/ijs.0.051664-0 23811135

[B30] Hao O. J. Chen J. M. Huang L. Buglass R. L. (1996). Sulfate-reducing bacteria. *Crit. Rev. Environ. Sci. Technol.* 26 155–187. 10.1080/10643389609388489

[B31] He Z. Zhou A. Baidoo E. He Q. Joachimiak M. P. Benke P. (2010). Global transcriptional, physiological and metabolite analyses of the responses of *Desulfovibrio vulgaris* Hildenborough to salt adaptation. *Appl. Environ. Microbiol.* 76 1574–1586. 10.1128/AEM.02141-09 20038696 PMC2832388

[B32] Kapatral V. Anderson I. Ivanova N. Los T. Lykidis A. Bartman A. (2005). Genome sequence and analysis of the oral bacterium *Fusobacterium nucleatum* strain ATCC 25586. *J. Bacteriol.* 184 2005–2018. 10.1128/JB.184.7.2005 11889109 PMC134920

[B33] Keller K. L. Rapp-Giles B. J. Semkiw E. S. Porat I. Brown S. D. Wall J. D. (2014). New model for electron flow for sulfate reduction in *Desulfovibrio alaskensis* G20. *Appl. Environ. Microbiol.* 80 855–868. 10.1128/AEM.02963-13 24242254 PMC3911205

[B34] Keller K. L. Wall J. D. (2011). Genetics and molecular biology of the electron flow for sulfate respiration in *Desulfovibrio*. *Front. Microbiol.* 2:135. 10.3389/fmicb.2011.00135 21747813 PMC3129016

[B35] Klindworth A. Pruesse E. Schweer T. Peplies J. Quast C. Horn M. (2013). Evaluation of general 16S ribosomal RNA gene PCR primers for classical and next-generation sequencing-based diversity studies. *Nucleic Acids Res.* 41:e1. 10.1093/nar/gks808 22933715 PMC3592464

[B36] Klouche N. Basso O. Lascourreges J.-F. Cayol J.-L. Thomas P. Fauque G. (2009). Desulfocurvus vexinensis gen. nov., sp. nov., a sulfate-reducing bacterium isolated from a deep subsurface aquifer. *Int. J. Syst. Evol. Microbiol.* 59 3100–3104. 10.1099/ijs.0.010363-0 19643880

[B37] Kondo R. Nedwell D. B. Purdy K. J. De Queiroz Silva S. (2004). Detection and enumeration of sulphate-reducing bacteria in estuarine sediments by competitive PCR. *Geomicrobiol. J.* 21 145–157. 10.1080/01490450490275307

[B38] Korte H. L. Fels S. R. Christensen G. A. Price M. N. Kuehl J. V. Zane G. M. (2014). Genetic basis for nitrate resistance in *Desulfovibrio* strains. *Front. Microbiol.* 5:153. 10.3389/fmicb.2014.00153 24795702 PMC4001038

[B39] Korte H. L. Saini A. Trotter V. V. Butland G. P. Arkin A. P. Wall J. D. (2015). Independence of nitrate and nitrite inhibition of *Desulfovibrio vulgaris* hildenborough and use of nitrite as a substrate for growth. *Environ. Sci. Technol.* 49 924–931. 10.1021/es504484m 25534748

[B40] Kovarova-Kovar K. Egli T. (1998). Growth kinetics of suspended microbial cells: from single-substrate-controlled growth to mixed-substrate kinetics. *Microbiol. Mol. Biol. Rev.* 62 646–666. 9729604 10.1128/mmbr.62.3.646-666.1998 PMC98929

[B41] Lambo A. J. Noke K. Larter S. R. Voordouw G. (2008). Competitive, microbially-mediated reduction of nitrate with sulfide and aromatic oil components in a low-temperature, western Canadian oil reservoir. *Environ. Sci. Technol.* 42 8941–8946. 10.1021/es801832s 19192822

[B42] Lane D. J. (1991). “16S/23S rRNA sequencing,” in *Nucleic Acid Techniques in Bacterial Systematics*, eds Stackebrandt E. Goodfellow M. (Chichester: Wiley), 115–175.

[B43] Leschine S. Paster B. J. Canale-Parola E. (2006). “Free-living saccharolytic *Spirochetes*: the genus *Spirochaeta*,” in *The Prokaryotes*, eds Dworkin M. Falkow S. Rosenberg E. Schleifer K. H. Stackebrandt E. (New York, NY: Springer), 195–210.

[B44] Magot M. Ravot G. Campaignolle X. Ollivier B. Patel B. K. Fardeau M. L. (1997). *Dethiosulfovibrio peptidovorans* gen. nov., sp. nov., a new anaerobic, slightly halophilic, thiosulfate-reducing bacterium from corroding offshore oil wells. *Int. J. Syst. Bacteriol.* 47 818–824. 10.1099/00207713-47-3-818 9226912

[B45] Martin K. J. Picioreanu C. Nerenberg R. Dame N. Hall F. Dame N. (2015). Assessing microbial competition in a hydrogen-based membrane biofilm reactor (MBfR) using multidimensional modeling. *Biotechnol. Bioeng.* 112 1843–1853. 10.1002/bit.25607 25854894

[B46] Mehta-Kolte M. G. Loutey D. Wang O. Youngblut M. D. Hubbard C. G. Wetmore K. M. (2017). Mechanism of H2S oxidation by the dissimilatory perchlorate-reducing microorganism Azospira suillum PS. *MBio* 8 1–16. 10.1128/mBio.02023-16 28223460 PMC5358917

[B47] Meyer B. Kuehl J. Deutschbauer A. M. Price M. N. Arkin A. P. Stahl A. (2013). Variation among *Desulfovibrio* species in electron transfer systems used for syntrophic growth. *J. Bacteriol.* 195 990–1004. 10.1128/JB.01959-12 23264581 PMC3571329

[B48] Nemati M. Jenneman G. E. Voordouw G. (2001). Mechanistic study of microbial control of hydrogen sulfide production in oil reservoirs. *Biotechnol. Bioeng.* 74 424–434. 10.1002/bit.1133 11427944

[B49] Oremland R. S. Capone D. G. (1988). Use of ”Specific” inhibitors in biogeochemistry and microbial ecology. *Adv. Microb. Ecol.* 10 285–383.

[B50] Plugge C. M. Zhang W. Scholten J. C. M. Stams A. J. M. (2011). Metabolic flexibility of sulfate-reducing bacteria. *Front. Microbiol.* 2:81. 10.3389/fmicb.2011.00081 21734907 PMC3119409

[B51] Postgate J. (1949). Competitive inhibition of sulfate reduction by selenate. *Nature* 164 670–671.

[B52] Postgate J. R. (1952). Competitive and non-competitive inhibitors of bacterial sulphate reduction. *J. Gen. Micriobiol* 6 128–142. 10.1099/00221287-6-1-2-128 14927859

[B53] Rittman B. E. McCarty P. L. (2001). *Environmental Biotechnology: Principles and Applications*. New York, NY: McGraw-Hill.

[B54] Roberts D. J. Nica D. Zuo G. Davis J. L. (2002). Quantifying microbially induced deterioration of concrete: initial studies. *Int. Biodeterior. Biodegrad.* 49 227–234. 10.1016/S0964-8305(02)00049-45

[B55] Schloss P. D. Westcott S. L. Ryabin T. Hall J. R. Hartmann M. Hollister E. B. (2009). Introducing mothur: open-source, platform-independent, community-supported software for describing and comparing microbial communities. *Appl. Environ. Microbiol.* 75 7537–7541. 10.1128/AEM.01541-09 19801464 PMC2786419

[B56] Smith H. L. Waltman P. E. (1995). *The Theory of the Chemostat: Dynamics of Microbial Competition*. Cambridge, NY: Cambridge University Press.

[B57] Spence C. Whitehead T. R. Cotta M. A. (2008). Development and comparison of SYBR Green quantitative real-time PCR assays for detection and enumeration of sulfate-reducing bacteria in stored swine manure. *J. Appl. Microbiol.* 105 2143–2152. 10.1111/j.1365-2672.2008.03900.x 19120660

[B58] Sun J. Hu S. Sharma K. R. Ni B. Yuan Z. (2014). Stratified microbial structure and activity in sulfide- and methane- producing anaerobic sewer biofilms. *Appl Env. Microbiol* 80 7042–7052. 10.1128/AEM.02146-14 25192994 PMC4249016

[B59] Surkov A. V. Dubinina G. A. Lysenko A. M. Glöckner F. O. Kuever J. (2001). *Dethiosulfovibrio russensis* sp. nov., Dethiosulfovibrio marinus sp. nov. and Dethiosulfovibrio acidaminovorans sp. nov., novel anaerobic, thiosulfate- and sulfur-reducing bacteria isolated from “Thiodenron” sulfur mats in differ. *Int. J. Syst. Evol. Microbiol.* 51 327–337. 10.1099/00207713-51-2-327 11321077

[B60] Takii S. Hanada S. Tamaki H. Ueno Y. Sekiguchi Y. Ibe A. (2007). Dethiosulfatibacter aminovorans gen. nov., sp. nov., a novel thiosulfate-reducing bacterium isolated from coastal marine sediment via sulfate-reducing enrichment with Casamino acids. *Int. J. Syst. Evol. Microbiol.* 57 2320–2326. 10.1099/ijs.0.64882-0 17911304

[B61] Voordouw G. Grigoryan A. A. Lambo A. Lin S. Park H. S. Jack T. R. (2009). Sulfide remediation by pulsed injection of nitrate into a low temperature Canadian heavy oil reservoir. *Environ. Sci. Technol.* 43 9512–9518. 10.1021/es902211j 20000549

[B62] Whitman W. B. Bowen T. L. Boone D. R. (2014). “The methanogenic bacteria,” in *The Prokaryotes*, eds Rosenberg E. DeLong E. F. Lory S. Stackebrandt E. Thompson Berlin F. (Heidelberg: Springer).

[B63] WHO (2000). *Hydrogen Sulfide. 1–7.* http://www.euro.who.int/__data/assets/pdf_file/0019/123076/AQG2ndEd_6_6Hydrogensulfide.PDF

[B64] Widdel F. Bak F. (1992). “Gram-negative mesophilic sulfate-reducing bacteria,” in *The Prokaryotes*, eds Balows A. Trüper H. G. Dworkin M. Harder W. Schleifer K. H. (New York, NY: Springer).

[B65] Zhou C. Zhou Y. Rittmann B. E. (2017). Reductive precipitation of sulfate and soluble Fe(III) by Desulfovibrio vulgaris ?: electron donor regulates intracellular electron flow and nano-FeS crystallization. *Water Res.* 119 91–101. 10.1016/j.watres.2017.04.044 28436827

