# Supplementary figures and images for: Resistance and Resilience of Sulfidogenic Communities in the Face of the Specific Inhibitor Perchlorate
Source: Front Microbiol. 2019 Apr 2;10:654. doi: 10.3389/fmicb.2019.00654 (PMC6454106; doi:10.3389/fmicb.2019.00654)

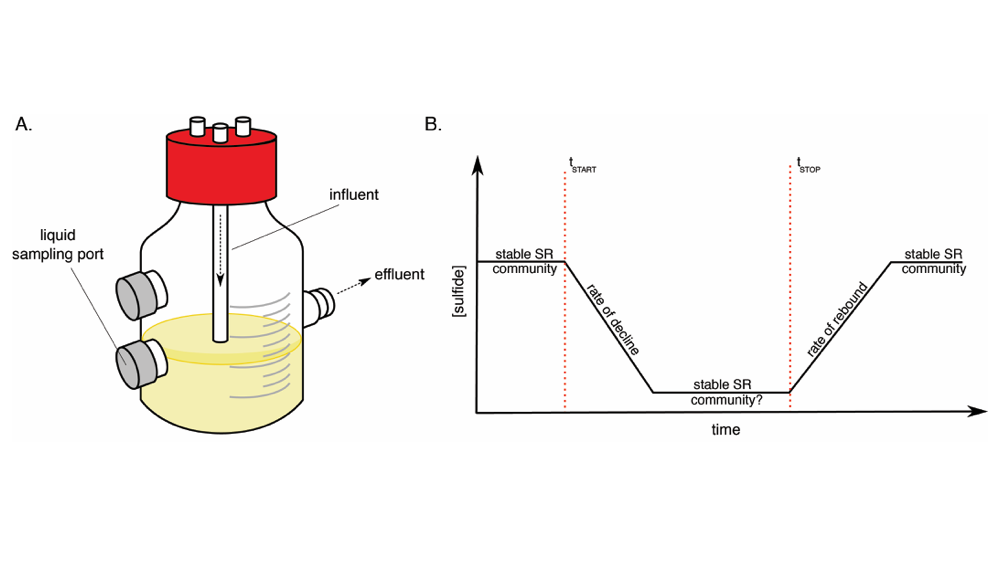

Supplement: FIGURE S1 — (A) Chemostat design: anaerobic media (influent) is pumped in; effluent (waste culture) drains passively. A culture of approximately 125 ml is maintained and sampled through the sampling port. (B) Experimental design: communities are allowed to reach stable sulfide production before treatment with perchlorate (tSTART). Various chemical parameters are monitored during inhibition and once inhibition levels off, treatment is stopped (tSTOP) and rebound is monitored. [file Image_1.TIFF]

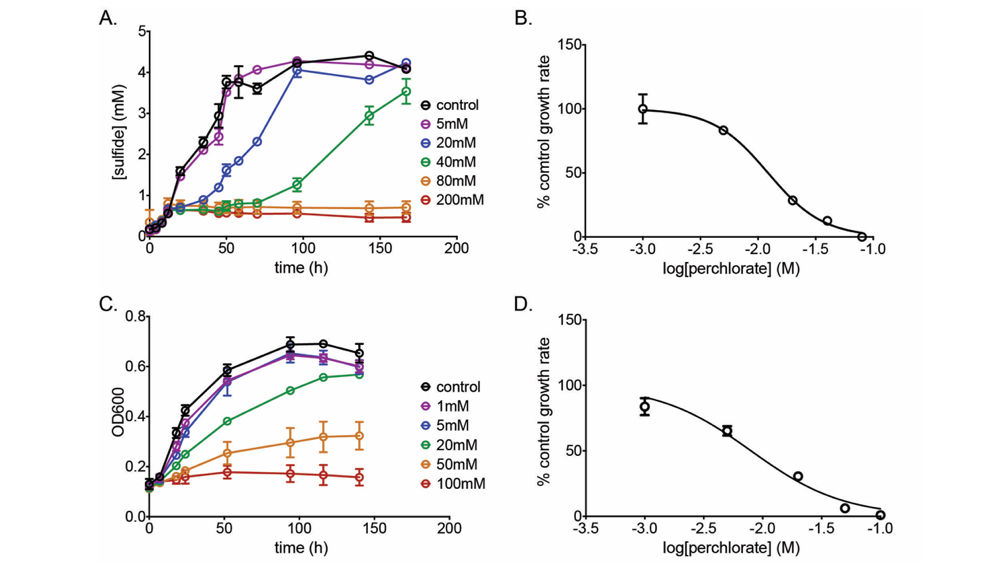

Supplement: FIGURE S2 — Growth curves of (A) community sulfide production and (C) Desulfovibrio sp. BMSR, at various perchlorate concentrations. Perchlorate was spiked in at 12 and 7 h respectively. Data generated was used to construct dose-response/inhibition curves for (B) community SRM and (D) D. sp. BMSR. Error bars represent SD of three replicates. [file Image_2.TIFF]

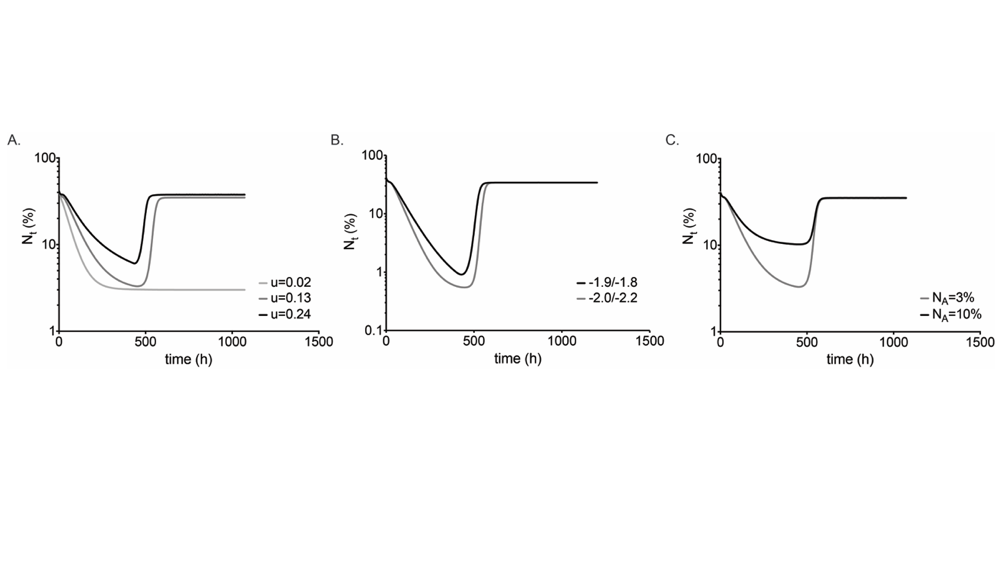

Supplement: FIGURE S3 — Model predictions for the effect of (A) increasing μ, (B) changing the dose-response/inhibition curve (IC50/Hillslope parameters) and (C) increasing Nt, while keeping all other model parameters constant. [file Image_3.TIFF]

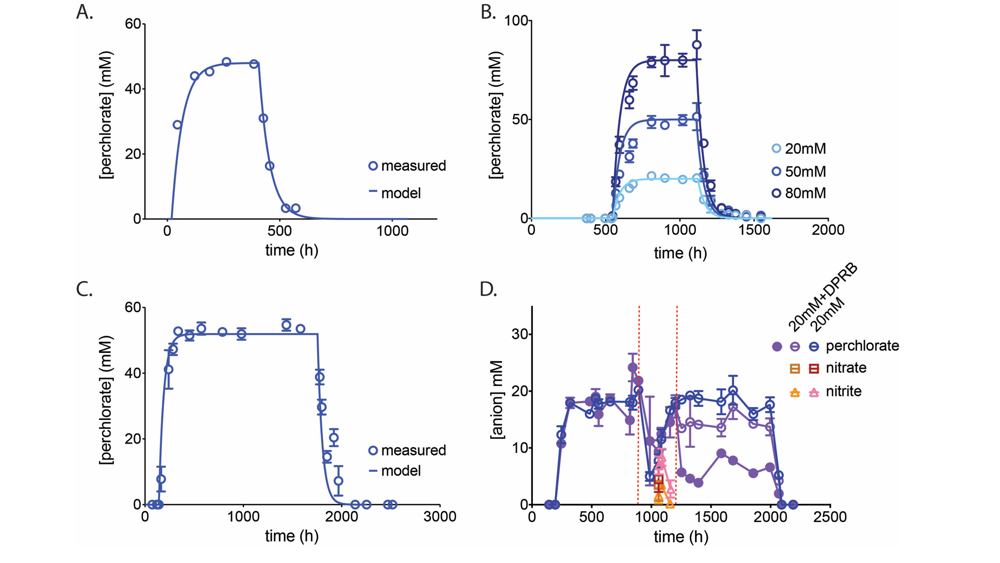

Supplement: FIGURE S4 — Perchlorate concentrations (circles– measured, solid line – modeled) for (A) initial chemostat run at 50 mM, (B) subsequent run at 20, 50, and 80 mM, and (C) surface-attachment run at 50 mM. (D) Measured perchlorate concentrations in DPRB-amendment run, for chemostats treated with 20 mM perchlorate (blue symbols), and 20 mM perchlorate and DPRB (purple symbols). The latter treatment is split into replicates 1–2 (open symbols) and replicate 3 (closed symbols). Nitrate and nitrite concentrations are also given, for the brief period of nitrate treatment, indicated by dashed red lines. Error bars represent SD. [file Image_4.TIFF]

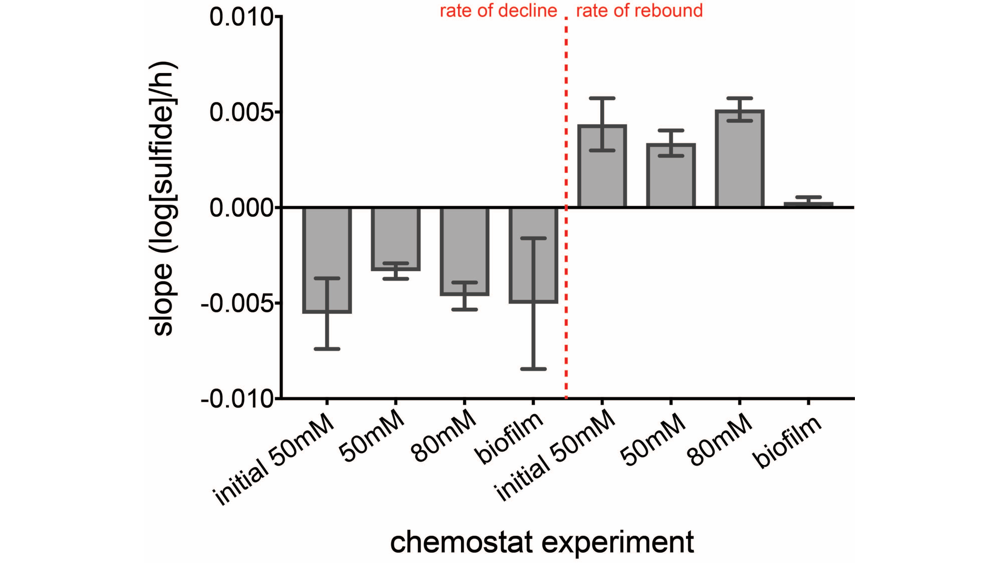

Supplement: FIGURE S5 — Slope of the linear regression fit to log transformed sulfide data during the period of inhibition and the period of rebound for each respective chemostat experiment. Bars represent the 95% CI. [file Image_5.TIFF]

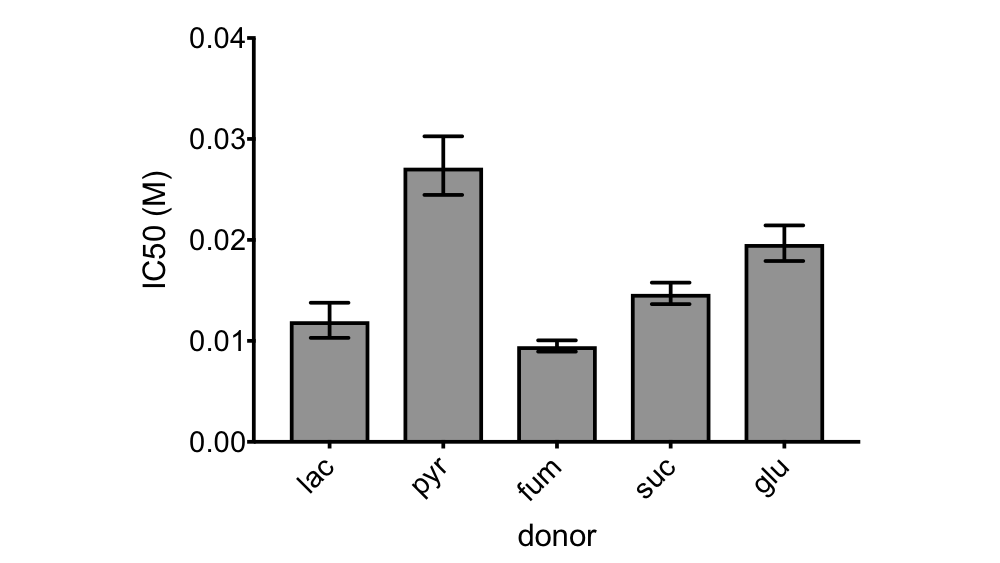

Supplement: FIGURE S6 — IC50 measurements for Desulfovibrio sp. BMSR on different donors: lac, sodium lactate; pyr, sodium pyruvate; fum, sodium fumarate; suc, sodium succinate; and glu, glucose. Error bars represent the 95% CI of the IC50 estimate. [file Image_6.TIFF]

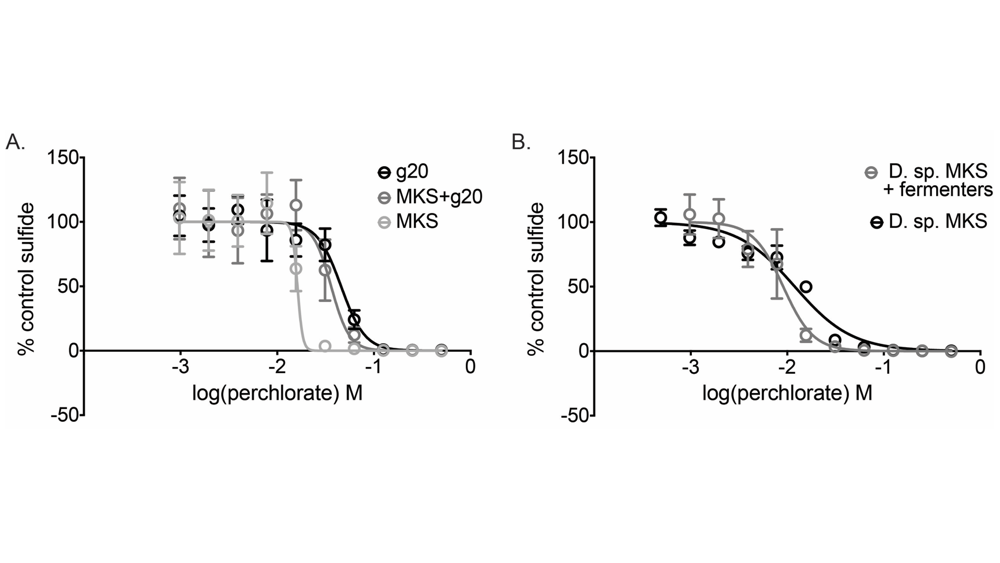

Supplement: FIGURE S7 — Dose-response/inhibition curves for (A) Desulfovibrio alaskensis G20 (black circles), Desulfovibrio sp. BMSR (light gray circles) and a co-culture of both organisms (gray circles) and (B) Desulfovibrio sp. BMSR in pure culture (black circles) and co-cultured with a mixed community of fermenters (gray circles). Error bars represent the SD of four replicates. [file Image_7.TIFF]
